# A metaproteomic analysis of the piglet fecal microbiome across the weaning transition

**DOI:** 10.3389/fmicb.2025.1504433

**Published:** 2025-05-02

**Authors:** Israel Rivera, KaLynn Harlow, Robert N. Cole, Robert O’Meally, Wesley Garrett, Weili Xiong, William Oliver, James E. Wells, Katie Lynn Summers, Nisan Chhetri, Olga Postnikova, Lea Rempel, Matt Crouse, Bryan Neville, Cary Pirone Davies

**Affiliations:** ^1^Animal Biosciences and Biotechnology Laboratory, Beltsville Agricultural Research Center, ARS, U.S. Department of Agriculture, Beltsville, MD, United States; ^2^Oak Ridge Institute for Science and Education, Agricultural Research Service Participation Program, Oak Ridge, TN, United States; ^3^Department of Biological Chemistry, School of Medicine, Johns Hopkins University, Baltimore, MD, United States; ^4^U.S. Food and Drug Administration, Center for Food Safety and Applied Nutrition, College Park, MD, United States; ^5^U.S. Meat Animal Research Center, ARS, U.S. Department of Agriculture, Clay Center, NE, United States; ^6^Department of Computer Science, College of Engineering, North Carolina State University, Raleigh, NC, United States

**Keywords:** metaproteome, microbiome, weaning, digestion, swine

## Abstract

Microbiome analysis has relied largely on metagenomics to characterize microbial populations and predict their functions. Here, we used a metaproteomic analysis of the fecal microbiome in piglets before and after weaning to compare protein abundances as they pertain to microbial populations specific to either a milk- or plant-based diet. Fecal samples were collected from six piglets on the day of weaning and 4 weeks after transitioning to a standard nursery diet. Using the 12,554 protein groups identified in samples, we confirmed the shift in protein composition that takes place in response to the microbial succession following weaning and demonstrated the redundancy in metabolic processes between taxa. We identified taxa with roles as primary degraders based on corresponding proteins synthesized, thereby providing evidence for cross-feeding. Proteins associated with the breakdown of milk-specific carbohydrates were common among pre-weaned pigs, whereas the proteome of post-weaned piglets contained a greater abundance of proteins involved in the breaking down plant-specific carbohydrates. Furthermore, output revealed that production of propionate takes place via the propionaldehyde pathway in pre-weaned piglets, but changes to production via the succinate pathway in post-weaned piglets. Finally, a disproportionate quantity of carbohydrate-active enzymes (CAZymes) (~8%) were produced by fungi, which typically only represent ~0.1% of the microbiome taxa. Information gathered through this characterization of the metaproteome before and after weaning revealed important differences regarding the role of members in the microbial community, thereby providing information for the optimization of diets and products for both piglet and microbiome health.

## Introduction

Weaning is a stressful transition in pigs, and abrupt changes in nutrition, the environment, and social structure often result in reduced growth and performance (Campbell et al., 2013; Pluske et al., 2018). Post-weaned diarrhea, degradation of the gut epithelial barrier, and a decrease in the digestion and absorption of nutrients are common (Li et al., 2018; Tang et al., 2022). Post-weaned recovery lags in some piglets and may result in lifelong growth impairment (Kats et al., 2010). Traditionally, in-feed antibiotics were used to ease the weaning transition, but with current limitations on antibiotic usage (Maron et al., 2013), novel therapeutics are needed.

The gut microbiome is a collection of diverse bacteria, fungi, and eukaryotes which occur within the gastrointestinal tract (GI) and impact numerous aspects of host physiology including intestinal and digestive health. Diet is one of the principal factors which shapes microbiome community composition (Frese et al., 2015; Oliphant and Allen-Vercoe, 2019). At weaning, as the diet shifts from milk to a primarily plant-based ration, nutrients available for both the piglet and the microbial community change, and the microbiome undergoes a dramatic taxonomic and functional shift (Feehan et al., 2022; Arfken et al., 2020; Harlow et al., 2023) which can result in dysbiosis and can contribute to negative health outcomes (Tang et al., 2022; Guevarra et al., 2019). Optimizing microbiome functions across this transition, especially the digestion of nutrients and production of beneficial metabolites, could ease the weaning transition and improve piglet health and productivity.

Microbes contribute to the breakdown and absorption of all macronutrients (Li et al., 2018; Tang et al., 2022; Zheng et al., 2021) but play a particularly critical role in the degradation and metabolism of carbohydrates. Like other mammals, pigs produce a small number of enzymes which degrade carbohydrates, among them alpha-amylase, oligosaccharidases such as α-glucosidases, and in older pigs, sucrase (Drochner, 1993). However, they are not able to degrade many complex carbohydrates in the diet and are reliant on microbes in the large intestine to complete this process (Bach et al., 2013). Bacterial genomes encode many different carbohydrate-active enzymes (CAZymes), which cleave complex oligosaccharides into mono- or disaccharides. These simple sugars are then metabolized via glycolysis and subsequently fermented into various metabolites, the most abundant of which are the short chain fatty acids (SCFAs) acetate, butyrate, and propionate. SCFAs are critical for mammalian health, serving not only as energy sources for colonic cells in the large intestine, but also as mediators of energy homeostasis, immunity, and gut barrier integrity (Rechkemmer et al., 1988; Knudsen et al., 2012; Peng et al., 2009; Diao et al., 2019; Fukuda et al., 2011). The complete process of carbohydrate degradation and generation of SCFAs is accomplished by the concerted effort of the microbial community in a process called cross-feeding where the metabolic end-product from one member of the community becomes a substrate for a different member (Mataigne et al., 2021; Goyal et al., 2021; Culp and Goodman, 2023).

Despite growing evidence of the critical role of microbes in digestion and health, many mechanisms by which microbes contribute to piglet well-being after weaning remain unclear. To date, most microbiome studies rely on metagenomic or amplicon-based sequencing methods to catalogue the taxa present in a sample and predict their functions, or metatranscriptomics which describes the genes that are transcribed. However, these approaches do not account for post-transcriptional (Arraiano et al., 2010; Gottesman and Storz, 2011; Potts et al., 2017; Hausmann et al., 2021) and regulation mechanisms which bacteria employ in response to their environments (Macek et al., 2019). In this study we employed a quantitative metaproteomic approach to identify the microbial taxa responsible for carbohydrate metabolism and the generation of SCFAs in the piglet gut before and after weaning and highlight the carbohydrate digestive processes. An advantage of this metaproteomic approach is that it allows for the identification of actively expressed proteins and thus provides an accurate functional representation of cell metabolism. This information will facilitate the design of novel therapeutics which target the gut microbiome during the weaning transition to improve piglet health and performance.

## Materials and methods

### Animals

This animal study was reviewed and approved by the USDA-ARS Institutional Animal Care and Use Committee of the Meat Animal Research Center (Project # 133.0). Six piglets from the same litter were assessed from farrow through day 49 of life; piglets were weaned at day 21. Piglets were not provided with milk replacer/supplement or creep feed at any point throughout the experiment and were evaluated to be healthy, and no antibiotics, antifungals, or supplementary additives were administered to the piglets at any time. Piglets were housed in individual farrowing crates for the entirety of lactation. Post weaned piglets were co-housed in a single pen with their littermates. At weaning, piglets were given a commercially available transition starter diet for 2 days (Phase 1: 438-HE WOA Starter Feed (United Animal Health, Sheridan, IN), followed by two different phase diets (Phase 2: 2 days after weaning through 14 days after weaning (corn, soybean meal, soybean oil, and SS400 plus (United Animal Health, Sheridan, IN), a base nursery mix with milk proteins, lactose, and vit/min); Phase 3: 15 days after weaning through 28 days after weaning (corn, soybean meal, soybean oil, and HD 80 plus—base nursery mix with phytase, vit/min, and crystalline amino acids (no milk products). Diet was formulated to meet or exceed the National Research Council estimate of nutrient requirements (Council, N.R, 2012). At day 21 (immediately prior to weaning—pre-weaned) and day 49 (4 weeks after weaning—post-weaned), fecal samples were collected directly from the rectum of the piglets into a sterile basin and then transferred to a 2.0 ml tube and immediately frozen in liquid nitrogen until transport to the laboratory.

### Protein extraction

Proteins were extracted from fecal samples (microbial and piglet cells) following a modified protocol (Zhang et al., 2016; Zhang et al., 2018). In brief, feces (0.15 g) were vortexed in 1.5 ml ice cold PBS. The slurry was centrifuged (300 *g*, 4C, 5 min), and supernatant transferred into 5.0 ml tube. This process was repeated twice more, and supernatants were pooled. Combined supernatants were centrifuged (300 *g*, 5 min, 4°C) to remove debris and pellets were discarded. This was repeated three times, and supernatants were pooled. Final clarified supernatant was centrifuged (14,000 *g*, 20 min, 4°C). Pelleted cells were washed in PBS 3 times and incubated in 500 μl 4% SDS (w/v) in 50 mM Tris–HCl buffer (pH 8.0) with protease inhibitor cocktail (95°C for 10 min with gentle agitation in Eppendorf Thermomixer). After cooling, these first-step lysates were subjected to three ultrasonications (30 s each with 30 s intervals on ice) using Q125 Sonicator (Qsonica, LLC) with amplitude of 25%. Cell debris was removed via centrifugation (16,000 *g*, 4°C for 10 min), and proteins in the supernatant were reduced and alkylated to improve resolubilization. Proteins were precipitated using 5-fold volume acidified acetone/ethanol at −20°C for 2 h. Sample was centrifuged (16,000 *g*, 20 min, 4°C) to form a protein pellet. Pellet was washed with ice cold acetone three times and resolubilized in 2% SDS.

### Isobaric mass tag labeling and fractionation

Protein extracts were buffer exchanged using SP3 paramagnetic beads (GE Healthcare) (Hughes et al., 2019). Briefly, samples were rehydrated in 100 μl of 100 mM triethylammonium bicarbonate (TEAB) and disulfide bonds reduced with 10 μl of 50 mM dithiothreitol for 1 h at 60°C. Samples were cooled to room temperature and pH adjusted to ~7.5, followed by alkylation with 10 μl of 100 mM iodoacetamide in the dark at RT for 15 min. Next, 100 μg (2 μl of 50 μg/μl) SP3 beads were added to the samples, followed by 120 μl of 100% ethanol. Samples were incubated at RT with shaking for 5 min. Following protein binding, beads were washed with 180 μl 80% ethanol three times. Proteins were digested on-bead with trypsin/LysC (Pierce) at 37°C overnight (1 μg enzyme) in 100ul of 100 mM TEAB. The supernatant containing digested peptides was removed from the beads using a magnetic rack and placed in a separate 0.5 ml Eppendorf vial.

To normalize the protein amount across all samples, a nanodrop apparatus was used to estimate the peptide amount per sample by absorbance at 280 nm. It was found that the lowest amount of protein was 8.7 μg, so all other samples were adjusted to that amount by their calculated volume and dried. Prior labeling with Tandem Mass Tags (TMT), the volumes of all samples were brought up to 50 μl in 100 mM TEAB. Samples were randomized and labeled with TMTPro reagents (Lot WH324722) with one label per sample and allowed to react for 1 h. After reaction, samples were each quenched with 0.5% hydroxyl amine for 15 min before mixing. The mixed sample was dried in a speed vac and then reconstituted in 2 ml of 10 mM TEAB before being subjected to fractionation by basic pH reversed-phase liquid chromatography. Eighty-four fractions were collected and dried. The eighty-four fractions were then concatenated down to 24 fractions to ensure an even distribution of peptides of varied hydrophobicity. The 24 fractions were dried down again then brought up in 2% acetonitrile, 0.1% formic acid in a 96 autosampler well plate.

### Mass spectrometry analysis and data analysis

Peptides in each of the 24 fractions were analyzed by reversed-phase liquid chromatography tandem mass spectrometry on an Orbitrap-Fusion Lumos interfaced with an Easy-nLC1200 (ThermoFisher Scientific). Peptides were separated a on a 75 μm x 20 cm picofritted column in-house packed with 3 μm, 120 Å ReproSIL-Pur-120-C18-AQ (Dr. Maisch, ESI Source Solutions) using a 2–90% acetonitrile in 0.1% formic acid gradient over 90 min at 300 nL/min. Eluting peptides were ionized at 2.5 kV. Survey scans (MS) of precursor ions were acquired with a 2 s cycle time from 375–1,500 m/z at 120,000 resolutions at 200 m/z with automatic gain control (AGC) at 4e5 and a 50 ms maximum injection time (IT). Precursor ions were individually isolated within 0.5 m/z by data dependent monitoring and 15 s dynamic exclusion and fragmented using an HCD activation collision energy 38. Fragmentation spectra (MS/MS) were acquired starting from 120 m/z at 50,000 resolutions using a 1.25e5 AGC and 86 ms IT. The Tandem Mass Tags were visible in MS2 spectra in the mass range from 128–134 Daltons and correspond to the relative abundance of the original peptides across the 12 protein extracts.

The data from the 24 fractions were combined and searched using the Mascot search engine (Matrix Science Version 2.8.3) inside the Proteome Discoverer interface (Thermo Scientific Version 2.5) and filtered to a 5% False Discovery Rate using the Percolator algorithm. Data were searched against the custom database (see below) and normalized based on Total Peptide Amount in the Reporter Ion Quantifier node. Precursors with a co-isolation of 30% or higher were filtered out to mitigate interferences from coeluting peptides. Protein grouping followed methods in Proteome Discoverer, with proteins grouped based on shared peptides. Each protein group has at least one unique peptide. Protein abundances were calculated as the sum of reporter ion abundances from the unique peptides. Taxonomy was assigned to each group by least common ancestor (LCA). Significant differences in protein abundances across the two timepoints were calculated using linear models in the limma Bioconductor software package (Ritchie et al., 2015) in R (Team, R.C, 2014) using paired test and multiple test correction for a 5% False Discovery Rate. Proteins with an adj. *p*-value ≤ 0.05 were considered differentially abundant between pre- and post-weaned groups. Average Expression (AveExpr), t-statistic, p-value, adjusted p-value, and Bayes Factor (BF), were given in Supplementary Table S1 for all protein groups.

### Database construction

Databases were constructed based on the 16S and ITS identifications of bacteria and fungi present in prior work (Harlow et al., 2023), publicly available at NCBI repository, accession number PRJNA1020867.

As species level classifications of amplicon data are often unreliable, we utilized genus level identifications. For those genera with percent abundance values greater than 2%, the proteomes of four to six “representative genomes” as defined by RefSeq were randomly selected for inclusion in the database and downloaded from RefSeq. If “representative genomes” were not available for the genus, a proteome was randomly selected from “complete genomes.” We also included proteomes from six most abundant fungi in our dataset, *Talaromyces*, *Aspergillus*, *Candida*, *Meyerozyma*, *Kazachstania*, and *Fusarium*. A list of species and accessions of the proteomes are listed in Supplementary Table S2. The *Sus scrofa* proteome (Genbank GCF_000003025.6) was utilized as a pig database. Spectra were independently searched against both this microbial database (bacteria and fungi) and pig databases. Microbial database consisted of 536,463 proteins. An average of 31,699 spectra were processed into peak lists for each sample, and an average of 2,905 microbial and 660 pig PSMs were identified per sample (11% of all spectra were assigned to either microbial or pig sequences).

### Pathway analysis

All master protein sequences were annotated using the eggNOG-mapper web application (v.2.1.12, available at http://eggnog-mapper.embl.de/) keeping default parameters. Functional classifications were assigned using the resulting KEGG (Kyoto Encyclopedia of Genes and Genomes) orthology (KO) and pathways classifiers (Huerta-Cepas et al., 2019; Kanehisa, 2019). Selected protein groups were further evaluated using MetaCyc database of metabolic pathways and enzymes (Caspi et al., 2020).

SCFA pathways were determined based on KEGG pathways for acetyl-CoA butyrogenic pathway (butanoate metabolism (ko00650), succinate, acrylate and propanediol propiogenic pathways (propanoate metabolism ko00640), and acetogenesis for Wood-Ljungdahl pathway (KEGG Module: M00377 and pyruvate metabolism (KEGG pathway:map00620) as modified in Tanca et al. (2024). A small number of additional proteins unique to this dataset but not present in Tanca et al. (2024) were selected based on their direct involvement in SCFA synthesis. Proteins present in multiple SCFA pathways were aggregated into a single pathway labeled with all sub-pathway names (e.g., acetate/propionate/butyrate). The pathway differential abundance between groups was assessed by averaging each protein within a pathway (row-wise mean) and the overall mean was computed to represent the mean for the entire pathway. Statistical significance was calculated using the Wilcoxon Signed-Rank paired-test with the Bonferroni correction (*n* = 6; *p*-value ≤ 0.05 is significant).

### CAZyme analysis

CAZymes were identified using the online dbcan3 server with default parameters (Zheng et al., 2023). In brief, the following search algorithms, databases, and significance thresholds were utilized: HMMER against the dbCAN3 database (e-value < 1e-15, coverage > 0.35), DIAMOND against the CAZy database (e-value < 1e-10^2), and HMMER against the dbCAN-sub database (e-value < 1e-15, coverage > 0.35). CAZyme annotation was reported in Supplementary Table S1 if output from at least two of the three tools reached values above the threshold, but the majority of glycoside hydrolase annotations were supported by all three tools (156/170) and only those proteins are discussed in the text.

### Identification of SCFAs and other metabolites

To determine homology of identified proteins to enzymes of interest, reference enzyme sequences documented to confer biochemical activity were identified in the literature. These reference sequences were searched against identified proteins using blastp (pident 25%, qcov > 60%), followed by confirmation that the identified proteins in our dataset contained the same domain conferring biological activity as present in the reference sequence (CDD, conserved domain database) (Wang et al., 2023).

## Results

### Core piglet gut metaproteome before and after weaning

**Figure 1:**
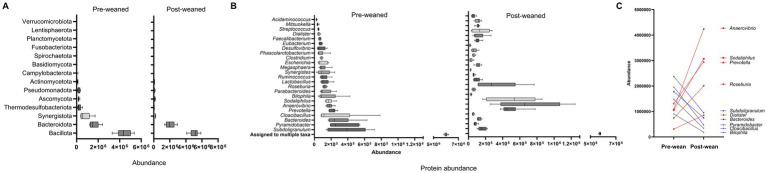
Protein abundance of the gut microbiome in pigs before and after weaning (*n* = 6). **(A)** Boxplot of top phyla ordered by increasing protein abundance. **(B)** Box plots of top genera ordered by increasing protein abundance at pre-weaning. **(C)** Connecting lines displaying the highest shift in protein abundance by genera during weaning. **Red** denotes an increase, and **blue** denotes a decrease in the abundance of protein groups after weaning.

We identified a total of 12,554 protein groups (Supplementary Table S1) in the piglet metaproteome. Of these, 3,334 proteins displayed greater abundance on day 21 (*p*-value < 0.05), whereas 2,614 proteins were more abundant on day 49. The remaining 6,606 proteins were detected at similar levels between pre- and post-weaned pigs. Of the 12,554 protein groups, 6,867 (55%) contained only a single protein and thus taxonomy was assigned at the genus level. The remaining 45% contained multiple proteins and taxonomy was assigned at various taxonomic levels using the least common ancestor approach (Supplementary Table S3). The most abundant protein groups corresponded to the phylum Bacillota, followed by Bacteroidota, regardless of time of sampling (Figure 1A). However, we observed marked differences in the protein distribution among genera between pre- and post-weaned pigs. In pre-weaned pigs, the most abundant protein groups corresponded to the genus *Subdoligranulum*, followed by *Pyramidobacter*, and *Bacteroides*. In contrast, the genera *Anaerovibrio*, *Sodaliphilus*, and *Prevotella*, were the most abundant in post-weaned pigs (Figure 1B). In addition to shifts among the most abundant taxa across weaning, proteins from *Cloacibacillus* and *Bilophila* also decreased from pre- to post wean and those from *Roseburia* and *Dialister* increased in abundance following weaning (Figure 1C).

### Functional annotation of the gut microbiome

Of the 12,554 protein groups inferred, 9,865 mapped to KEGG Ontology (KO; Supplementary Table S1) for a total of 1,855 KO functions and 355 KEGG pathways. The top protein groups identified include the translation elongation transport proteins (Elongation transport Ef Tu and Ef-G), multiple small subunit ribosomal proteins, and the molecular chaperones GroEL and DnaK (Supplementary Figure S1A). Carbon metabolism was the most abundant pathway and was represented by glycolysis/gluconeogenesis, amino sugars, fructose and mannose, starch and sucrose metabolism. Generation of the SCFAs acetate, butanoate and propanoate metabolism were also identified (Supplementary Figure S1B). Additional pathways include purine and pyrimidine metabolism, tRNA, transport, amino acid metabolism, and microbial quorum sensing and response to stimuli (two-component systems).

### Carbohydrate digestion: glycoside hydrolases (GHs), polysaccharide lyases, and glycolysis

**Figure 2:**
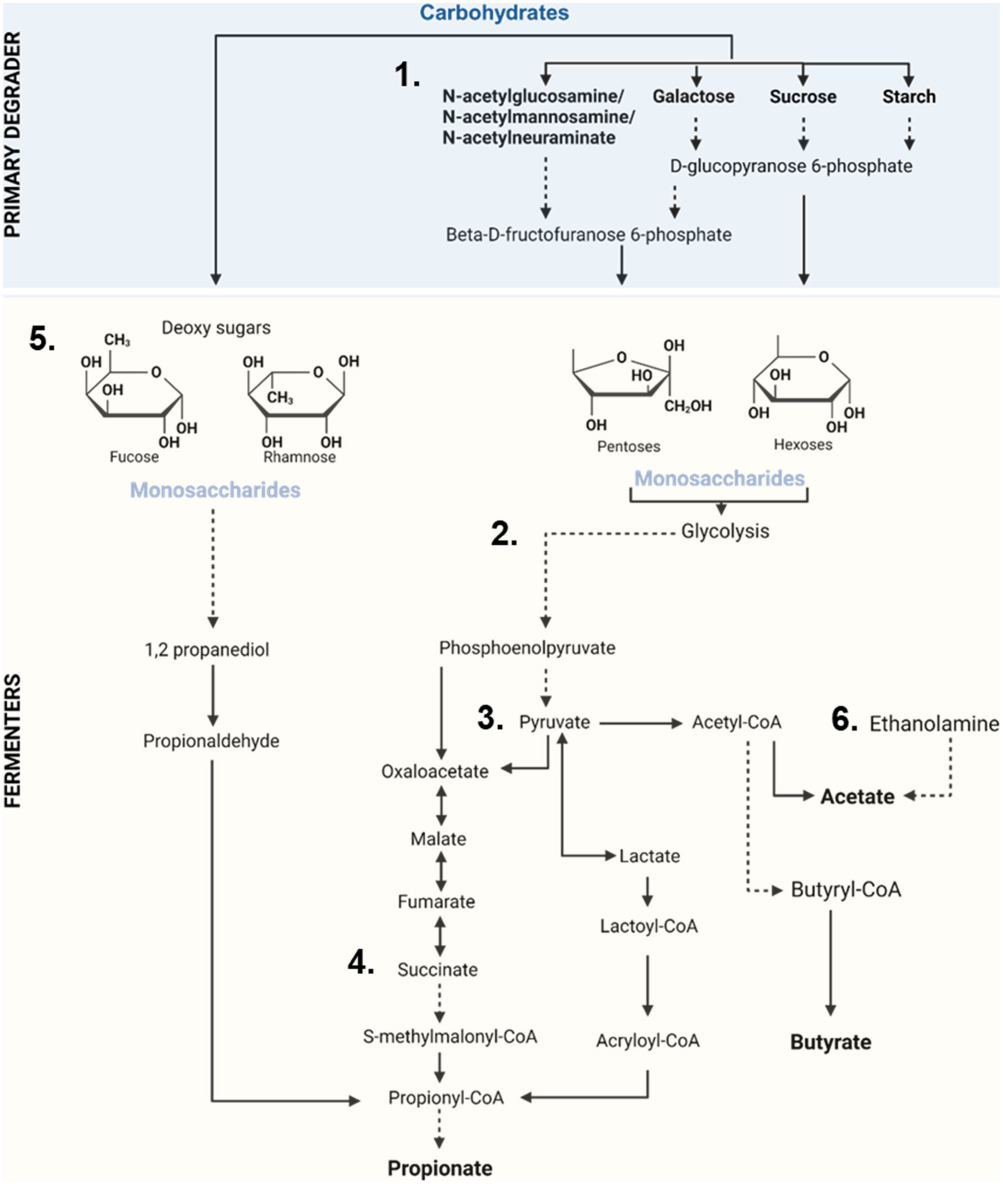
Overview of oligosaccharide degradation, carbohydrate utilization and production of SCFAs by the pig’s microbiota. (1) Complex non-digestible oligosaccharides are broken into simple monosaccharides units by the action of specialized microbes (Primary degraders). (2) Monosaccharides (hexoses/pentoses) then enter the glycolysis pathway where these are converted into phosphoenolpyruvate and ultimately into pyruvate by microbial fermenters. (3) Pyruvate then serves as the main precursor for the generation of short chain fatty acids (acetate, propionate, and butyrate). (4) Propionate production largely takes place via succinate. (5) Other sugars (like the deoxy sugars, fucose and rhamnose) can enter the propanediol pathway to generate propionate. (6) Production of acetate can also take place via the degradation of ethanolamine by specialized microbes.

**Figure 3:**
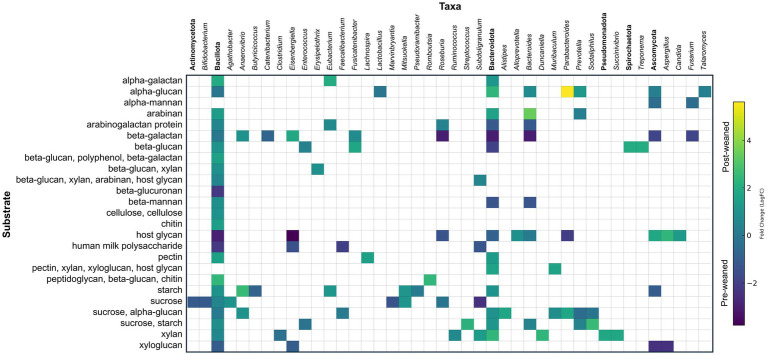
Taxonomic and abundance distribution of proteins involved in oligosaccharide digestion (CAZymes). Matrix of LogFC for CAZymes per taxon against their specific substrate for pre- and post-weaned pigs (*n* = 6). Protein groups assigned to the same genus that work on the same substrate were summed and represented by a single square. Annotations in bold denote phylum and represent the summed abundances of all lower taxonomic levels within that phylum.

Digestion of carbohydrates requires the enzymatic breakdown of dietary oligosaccharides into simpler units which are then channeled into the glycolytic pathway. This process requires the action CAZymes produced by primary degraders, especially glycoside hydrolases (GHs) and polysaccharide lyases (PLs) (Wardman et al., 2022) (Figure 2). In our analysis, 312 protein groups annotated as CAZymes (156 GHs, 2 PLs) were detected across both timepoints, with 112 of these annotated to a specific substrate (Figure 3, Supplementary Table S1). The abundances of 85 GHs differed significantly across timepoints. Six of the 85 were predicted to hydrolyze bonds of milk oligosaccharides (GH112) and were more abundant before weaning. These belonged to the genera *Faecalibacterium*, *Subdoligranulum*, *Eisenbergiella*, one to the class Clostridia, and two to the superkingdom Bacteria. Sixteen enzymes produced by members of the Bacillota and Bacteroidota phyla and the fungi *Aspergillus* and *Candida*, were predicted to degrade host glycans, likely mucins. Eight were significantly more abundant pre-weaned, whereas only one was more abundant post-weaned, from the genus *Bacteroides*. In contrast, 29 GHs predicted to degrade plant-derived carbohydrates including sucrose, starch, xylan, β-galactan, pectin, β-glucan, β-mannan, and cellulose were more abundant post-weaned than pre-weaned. However, eleven proteins predicted to degrade β-galactan, sucrose, starch, β-mannan, and xyloglucan were significantly more abundant in pre-weaned animals.

Some GH proteins groups contained only a single protein and were thus confidently assigned to the genus level (104/156). Of these, the genus *Bacteroides* produced the largest diversity of GHs (11), followed by *Agathobacter* (6), *Eisenbergiella* (6), *Prevotella* (6), *Parabacteroides* (5), *Sodaliphilus* (5), *Roseburia* (5), and *Subdoligranulum* (5). Of the GH protein groups which were assigned an LCA of phylum or below (142/156), a greater number were assigned to the phylum Bacillota (72) than Bacteroidota (52); however, a greater proportion of proteins synthesized by Bacteroidota were predicted by SignalP to be exported from the cell (Bacteroidota: 30/52 proteins; Bacillota: 13/72).

**Figure 4:**
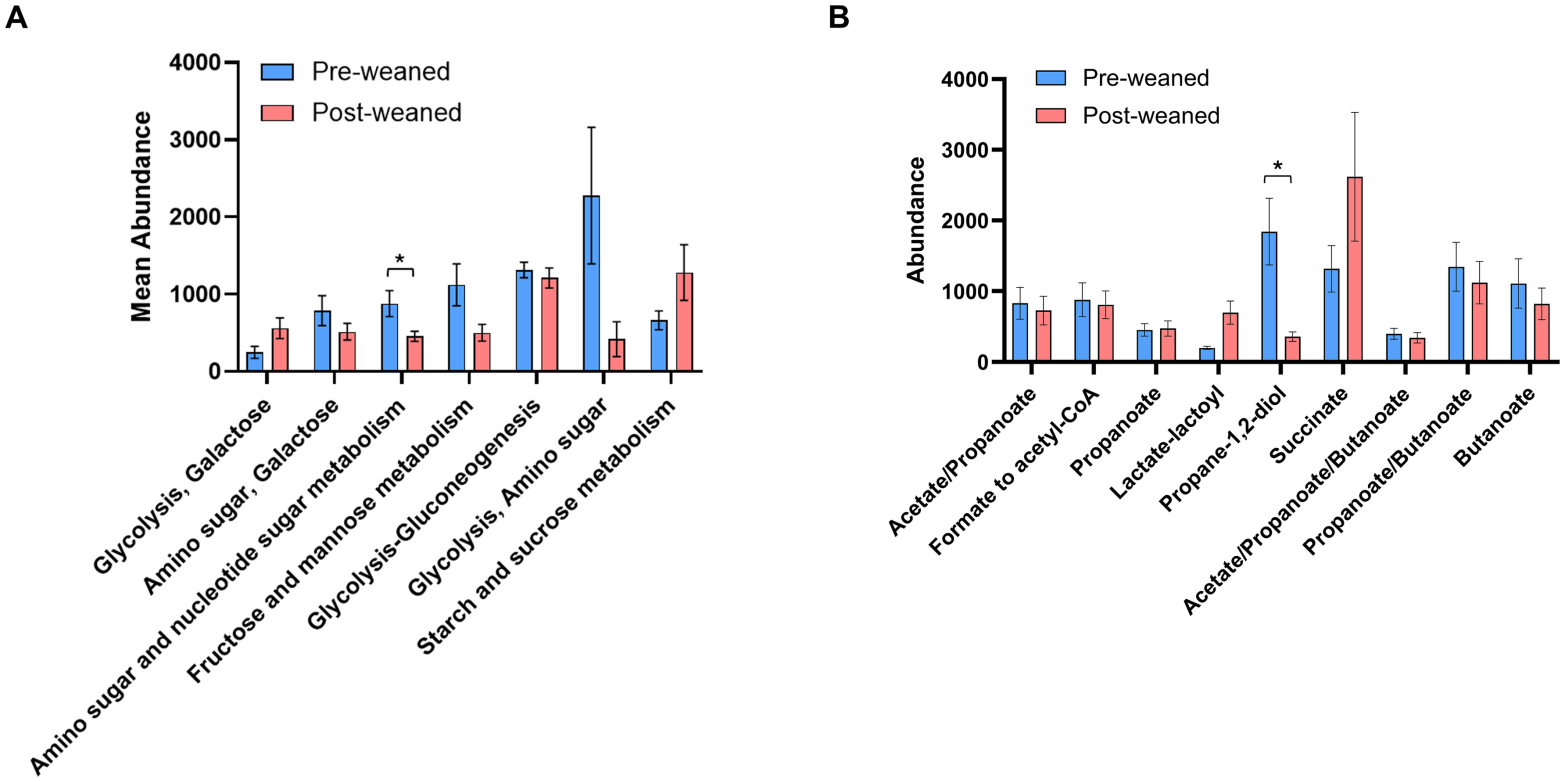
Carbohydrate and SCFA metabolism in pre- and post-weaned pigs. Differences in pathway abundance (KEGG pathways) between pre- and post-weaned groups (*n* = 6), were calculated using Wilcoxon paired test followed by Bonferroni correction. Proteins groups that could not be assigned to a single pathway, were joined into a single larger pathway. Asterisks * denote significant differences between groups (*p*-value ≤ 0.05). Mean abundance values are reported, and error bar indicates the standard error of the mean (SEM). **(A)** Comparison of carbohydrate metabolism between pre- and post-weaned pigs, only six pathways with the highest trending differences are shown. **(B)** Comparison of SCFA metabolism between pre- and post-weaned pigs.

**Figure 5:**
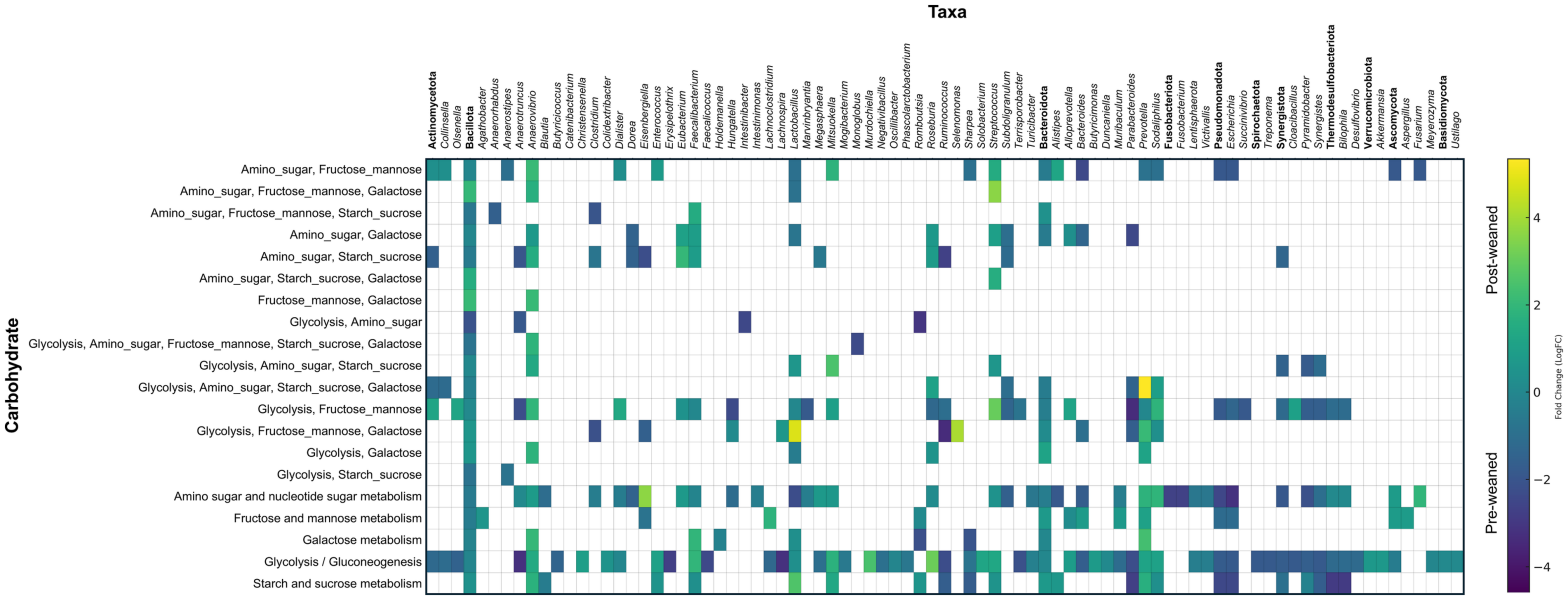
Taxonomic and abundance distribution of proteins involved in carbohydrate metabolism. Matrix of LogFC for carbohydrate associated proteins per taxon against their specific carbohydrate pathway (*n* = 6). Protein groups assigned to the same genus that work on the same substrate were summed and represented by a single square. Annotations in bold denote phylum and represent the summed abundances of all lower taxonomic levels within that phylum.

KEGG pathway analysis of carbohydrate metabolism also revealed that abundant pre-weaned microbial proteins were mainly associated with the breakdown of milk bioactive components (Supplementary Table S1) including those which metabolize milk oligosaccharides, fructose, mannose, amino sugars and nucleotide sugars (Figure 4A). Most of the pre-weaned proteins which degrade these carbohydrates were produced by members of the phyla Bacillota, including *Subdoligranulum, Anaerotruncus, Hungatella* and *Clostridium*, followed by Bacteroidota (Figure 5). These proteins mapped to the MetaCyc pathway “superpathway of N-acetylglucosamine, N-acetylmannosamine and N-acetylneuraminate degradation” (Caspi et al., 2014). N-acetylglucosamine comprises one of the repeating units commonly found in porcine milk oligosaccharides (PMOs) (Salcedo et al., 2016), and its breakdown results in the generation of β-D-fructofuranose 6-phosphate, a carbohydrate precursor to glycolysis. (Supplementary Figure S2A, Supplementary Table S1). Conversely, we observed evidence for the breakdown of plant-derived oligosaccharides in post-weaned pigs, although some were also observed before weaning. The most abundant proteins following weaning were associated with the metabolism of starch and sucrose (Figures 4A, 5), the main carbohydrates in feed (corn and soybean), which can be metabolized into D-glucopyranose 6-phosphate and β-D-fructofuranose 6-phosphate which serve as precursors to glycolysis metabolism. The predominant proteins involved in sucrose metabolism were from the order Selenomonadales (genera *Anaerovibrio* and *Mitsuokella*), followed by *Prevotella*, *Lactobacillus*, and *Sodaliphilus* (Supplementary Table S1).

After PMOs and/or plant oligosaccharides are converted into monosaccharides, they enter the glycolysis pathway. Regardless of timepoint, glycolysis was by far the most abundant pathway identified in our dataset at both timepoints (Supplementary Table S1). The genera responsible for producing glycolytic enzymes shifted over time with pre-weaned proteins attributed mainly to the orders *Eubacteriales*, *Bacteroidales*, *Enterobacterales*, *Synergistales*, and *Desulfovibrionales*, while post-weaned proteins were predominantly from the orders *Eubacteriales*, *Bacteroidales*, *Selenomonadales*, and *Lactobacillales*.

### Microbial contributions to SCFA production

#### Acetate production

Pyruvate, the main end-product of the glycolytic pathway, is converted into acetyl-CoA, which can then be hydrolyzed into acetate, or converted into propionate or butyrate through additional reactions. Differentiating which proteins are involved solely in acetate production can prove challenging. For example, proteins involved in acetate production were often linked to propionate and butyrate metabolism (Figure 4B, Supplementary Table S1). The abundances of acetate associated proteins, including those involved in the conversion of formate to acetyl-CoA via the Wood-Ljungdahl acetonic pathway (Ragsdale and Pierce, 2008), remained comparable between pre- and post-weaned pigs, and belonged mainly to the phyla Bacillota.

**Figure 6:**
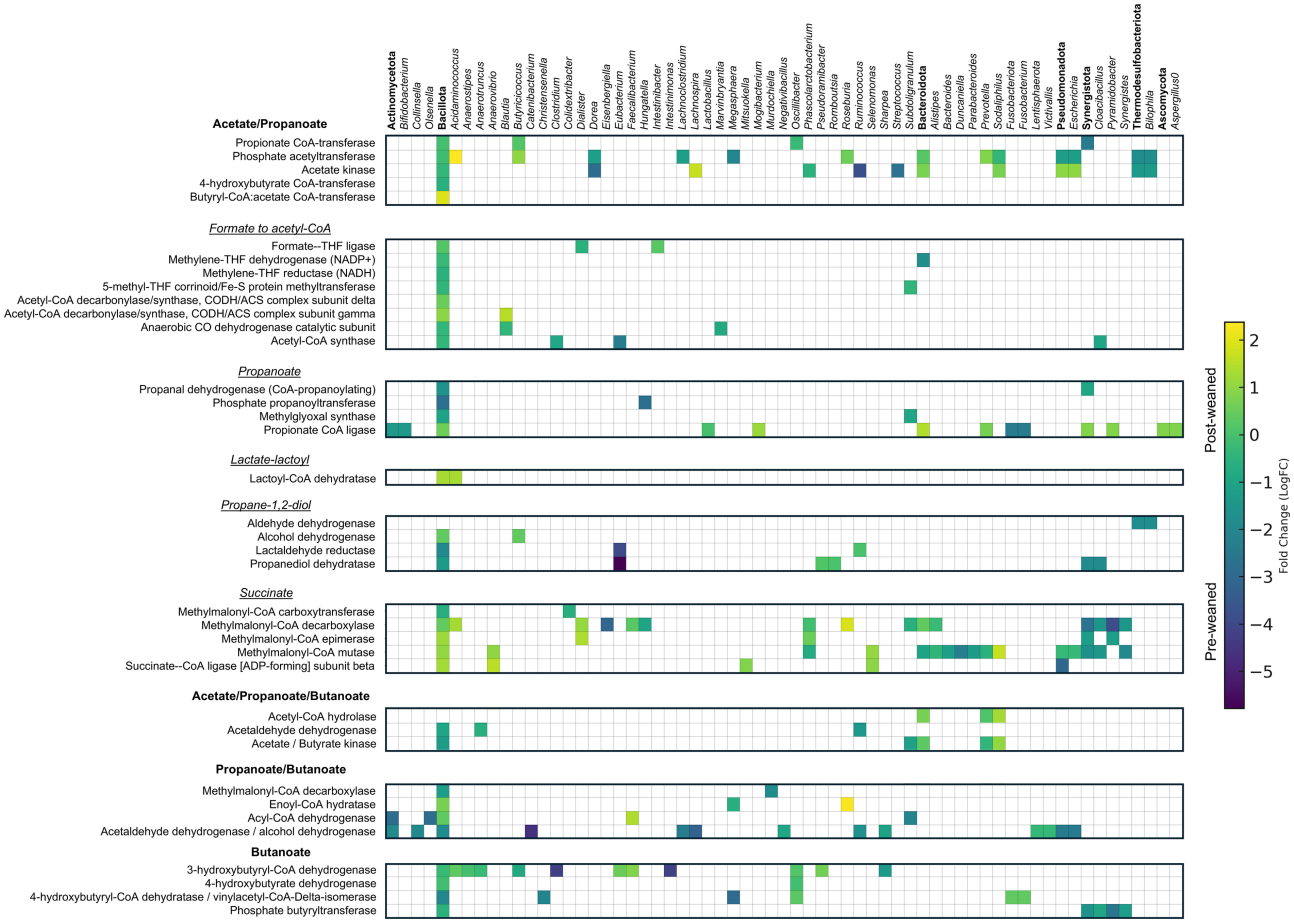
Taxonomic and abundance distribution of proteins involved in SCFA metabolism. Matrix of LogFC for SCFA proteins detected in pre- and post-weaned pigs (*n* = 6). Protein groups assigned to the same genus within the same pathway were summed and represented by a single square. Annotations in bold denote phylum and represent the summed abundances of all lower taxonomic levels within that phylum.

In pre-weaned pigs, proteins associated with acetate production were dominated by the genera *Ruminococcus, Dorea*, *Streptococcus*, *Megasphaera* and *Bilophila*, and by *Sodaliphilus*, *Lachnospira*, and *Acidaminococcus,* after weaning (Figure 6). We also identified proteins involved in the production of acetate from the utilization of ethanolamine in pre-weaned pigs. Ethanolamine is a component of eukaryotic membranes derived from phosphatidylethanolamine that can be converted to acetate by specialized microbes. We observed complete coverage of this pathway (Supplementary Figure S2B), with most of the protein groups (38%) belonging to the order Eubacteriales, followed by Synergistales.

We also determined which microbes contribute to the production of terminal enzymes for acetate production before and after weaning. These included phosphate acetyltransferase Pta (EC 2.3.1.8) which converts acetyl-CoA into acetyl phosphate, and acetate kinase AckA (EC 2.7.2.1) which converts acetyl phosphate to acetate. While these enzymes have additional roles in other metabolic processes, their primary function is tied to acetate production. Prior to weaning, the genera *Bacteroides*, *Lachnoclostridium* and *Dorea* were largely responsible for production of Pta, whereas the genera *Acidaminococcus*, *Prevotella* and *Butyricicoccus* were the main Pta producers after weaning. Production of AckA was mainly attributed to the genera *Subdoligranulum*, followed by *Ruminococcus* and *Dorea* before weaning and by the genera *Prevotella* and *Lachnospira* after weaning (Supplementary Table S1).

#### Propionate production

Propionate is the second most abundant SCFA after acetate, and its production mainly takes place through the metabolism of succinate (Xu et al., 2020). We evaluated the terminal enzymes leading to the formation of propionate, including the enzymes propionate kinase (EC 2.7.2.15), propionate CoA transferase (EC 2.8.3.1), and propionate CoA ligase (EC 6.2.1.17). Propionate kinase was more abundant in pre-weaned pigs (*Subdoligranulum* and *Ruminococcus*), while propionate CoA transferase was more abundant among post-weaned pigs (*Sodaliphilus* and *Eubacterium*).

**Figure 7:**
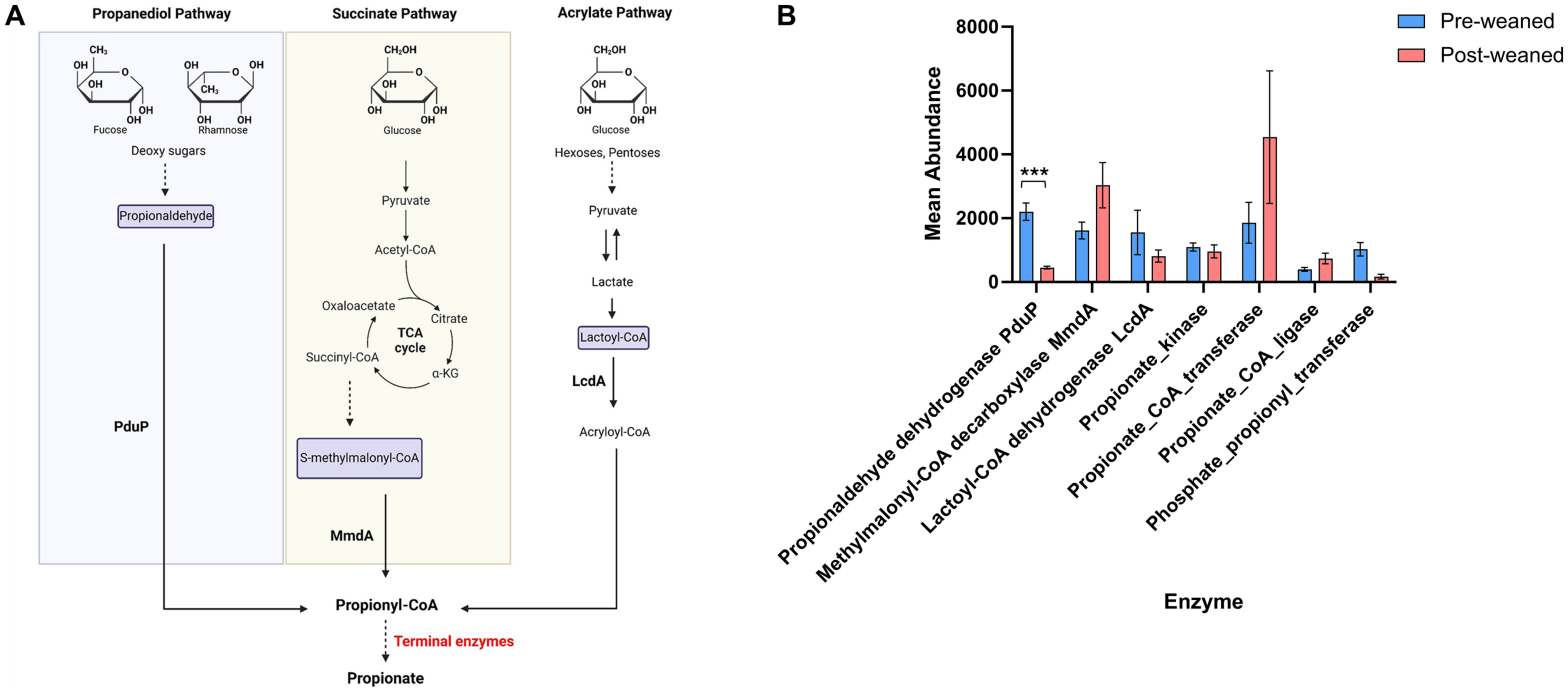
Propionate metabolism in pre- and post-weaned pigs. **(A)** Schematic of propionate production via the propanediol, succinate and acrylate pathways. **(B)** Protein homologous for propionaldehyde dehydrogenase (PduP), methylmalonyl-CoA decarboxylase (MmdA), lactoyl-CoA dehydrogenase (LcdA) and terminal enzymes (propionate kinase, propionate CoA transferase, propionate CoA ligase and Phosphate propionyl transferase) were compared between pre- and post-weaned pigs using Wilcoxon paired test followed multiple testing correction. Asterisks * denote significant differences between groups (p-value ≤ 0.05). Mean abundance values are reported, and error bar indicates the standard error of the mean (SEM).

Terminal enzymes involved in propionate production can also synthesize other SCFAs (e.g., acetate and butyrate). This introduces a challenge in identifying the production of propionate. To bypass this limitation, we determined which taxa produced enzymes which generate propionyl-CoA, the immediate precursor of propionate, as a proxy for propionate production (Reichardt et al., 2014). These include: (1) lactoyl-CoA dehydrogenase (lcdA) (EC 4.2.1.54) for the acrylate pathway, (2) propionaldehyde dehydrogenase (pduP) for the propanediol pathway, and the enzyme (3) methylmalonyl-CoA decarboxylase for the succinate pathway (Figure 7A). The most abundant enzyme in pre-weaned pigs was by far lcdA (Figure 7B), produced by *Lachnospira*, *Cloacibacillus*, *Escherichia* and *Lachnoclostridium*. In post-weaned pigs, the most abundant protein was methylmalonyl-CoA decarboxylase produced by *Dialister*, *Roseburia*, *Prevotella* and *Sodaliphilus* (Reichardt et al., 2014).

KEGG pathway analyses demonstrated an abundance of proteins associated with the propanoate pathway before weaning, and synthesized mainly by *Eubacterium*, *Cloacibacillus,* and *Bilophila*. After weaning, proteins associated with propionate production through the succinate pathway were elevated and produced by *Acidaminococcus*, *Roseburia*, *Sodaliphilus*, *Anaerovibrio* and *Dialister*.

#### Butyrate production

Pathway analysis identified a total of 106 proteins associated with butyrate production, of which the abundances of 50 were significantly different between pre- and post-weaned pigs (Supplementary Table S1). The most abundant proteins associated with butyrate metabolism belonged to the genera *Catenibacterium*, *Clostridium*, *Intestinimonas*, *Lachnospira*, and *Megasphaera*, at pre-weaned and the genera *Roseburia*, *Faecalibacterium*, and *Sodaliphilus* at post-weaned (Figure 6). However, many of these enzymes could also be involved in acetate or propionate metabolism.

To evaluate enzymes unique for butyrate metabolism, we assessed the abundance of terminal enzymes. The gut microbiota produces butyrate via four main pathways: (1) acetyl-CoA; (2) glutarate; (3) 4-aminobutyrate/succinate; and (4) lysine pathways, all of which converge at crotonyl-CoA. The rate limiting enzyme is butyryl-CoA dehydrogenase, bcd, which converts crotonyl-CoA to butyryl-CoA (Nielsen et al., 2009; Anand et al., 2016). The majority of butyrate is synthesized by the pyruvate and acetyl-coenzyme A (acetyl-CoA) pathways, resulting from the breakdown of non-digestible carbohydrates such as starch and xylan, but may also be synthesized from other metabolites such as lactate (Duncan et al., 2004). In this pathway, butyryl-CoA is converted to butyrate via either butyryl-CoA:acetate CoA transferase (but) (Trachsel et al., 2016), or phosphotransbutyrylase and butyrate kinase (buk) (EC 2.7.2.7) (Louis et al., 2004). We detected homologues of three terminal enzymes from two of the four pathways which produce butyrate (Supplementary Table S1), acetyl-CoA (but and buk), and lysine (butyryl-CoA:acetoacetate CoA transferase (atoAD)(EC 2.8.3.-). Four protein groups show homology to (but), four to buk, and two to atoAD (Cary et al., 1990). We did not detect homologues to butyryl-CoA:4-hydroxybutyrate CoA transferase (4hbt)(EC 2.8.3.-) (Louis et al., 2004). Homologues of butyryl-CoA:acetate CoA transferase (but) were produced by the genera *Pseudoramibacter*, *Roseburia*, and *Eubacterium*. Butyrate kinase (buk) is highly similar to acetate kinase (Vital et al., 2014). We identified 35 proteins which fit BLAST and CDD selection criteria for buk, but phylogenic assessment determined only four to be true homologues (data not shown). One buk homologue produced by the class Clostridia was higher pre-weaned, while buk proteins from *Sodaliphilus* were elevated (although not significant, adj. *p*-value = 0.056) post-weaned. We also detected a single homologue of butyryl-CoA:acetoacetate CoA transferase (atoA), which was detected from *Eubacterium*, while atoD was detected from *Romboutsia*. In total, we detected 10 unique terminal enzyme protein groups.

## Discussion

In this study, we report for the first time a temporal analysis of the fecal metaproteome in young piglets across the weaning transition. To our knowledge, only one metaproteomic analysis has been reported in young pigs (Saenz et al., 2021). However, that study was limited to mucosa and digesta from the jejunum and ileum of piglets from one time point, whereas the use of fecal samples in the current study allowed piglets to serve as their own controls to demonstrate the microbial variations that take place throughout the weaning transition.

Carbohydrates are the most abundant energy source in the swine diet and constitute 60–70% of total energy intake (Knudsen et al., 2016). Pigs produce a small number of carbohydrate-degrading enzymes, among them alpha-amylase, oligosaccharides such as α-glucosidases, and in older pigs, sucrase (Drochner, 1993). These enzymes assist in digestion of carbohydrates in the small intestine where they are readily absorbed. However, many dietary oligosaccharides pass undigested into the large intestine where they are degraded by the microbial community. CAZymes first catabolize these complex poly- and oligosaccharides into simple sugars that are then metabolized during glycolysis and finally fermented into SCFAs (Figure 2). These critical metabolites serve as an energy supply to colonocytes (Lauridsen, 2020) and may account for up to 25% of the host daily energy requirement intake (Holman et al., 2022).

### Microbial adaptations to dietary shifts

Transition from milk to a plant-based diet at weaning constitutes an abrupt change in the carbohydrate profile of the piglet diet (Frese et al., 2015; Amat et al., 2020). Lactose and porcine milk oligosaccharides (PMOs) constitute most of the carbohydrates available in milk (Knudsen et al., 2016; Gormley et al., 2024). Lactose is easily digested by the host, whereas PMOs consist of diverse structures of 3 to 20 monosaccharides including hexoses, D-glucose, D-galactose, N-acetylglucosamine, L-fucose, and N-acetylneuraminic acid (sialic acid) (Salcedo et al., 2016; Tao et al., 2010). After weaning, the majority of the diet (60–70%) is based on plant carbohydrates (Knudsen et al., 2012) from commercial diets containing corn (with soluble sucrose) (Knudsen, 1997), and soybean meal (with sucrose, as well as the nondigestible oligosaccharides stachyose and raffinose) (Hsu et al., 1973).

#### Pre-weaned CAZyme profile

The breakdown of milk oligosaccharides is accomplished through the concerted activities of enzymes from multiple GH families including sialidases, fucosidases, N-acetyl-β-hexosaminidases, and β-galactosidases (Derensy-Dron et al., 1999; El-Deep et al., 2021; Minamida et al., 2005). In both CAZyme and KEGG analyses, we identified high levels of proteins involved in the metabolism of PMOs among pre-weaned animals, including β-hexosaminidases, fucosidases, and 1,3-beta-galactosyl-N-acetylhexosamine phosphorylase, which cleaves the linkage between galactose and N-acetyl-D-glucosamine residues in MOs (Derensy-Dron et al., 1999) and was among the most abundant proteins before weaning. Taxa responsible for the breakdown of PMOs include *Faecalibacterium*, *Subdoligranulum*, *Eisenbergiella*, the family *Lachnospiraceae,* and the class Clostridia. The structures of mucin proteins are similar to those of PMOs, as mucins are decorated with o-glycosylated carbohydrates composed of N-acetyl-glucosamine, N-acetyl-galactosamine, and galactose cores which may be further modified by sulfate, fucose, and sialic acid residues (Pluske et al., 2018; Vasquez et al., 2022; Mahmud et al., 2023; Shen et al., 2022; Holmes et al., 2020). Thus, it is possible that the GH enzymes predicted to degrade PMOs could also degrade mucins (Guevarra et al., 2018; Lu et al., 2022), which has been previously reported for enzymes from *Akkermansia muciniphila* in the human microbiome (Brinsmade and Escalante-Semerena, 2004). Mucinolytic activity would explain the presence of these proteins in the microbiomes of post-weaned pigs, as the degradation of mucosal mucins could serve as a nutrient source for microbes (Garsin, 2010).

#### Post-weaned CAZyme profile

Following weaning, the microbiome adapted to a plant-based diet. We observed a greater abundance of GHs predicted to degrade various plant carbohydrates including sucrose, starch, cellulose, xylan, β-galactans, and arabinan. GHs that degrade cell wall components (e.g., glucans, α-glucans, β-glucans, and chitin) found in plants, fungi, lichens, and/or bacteria, were also more abundant after weaning. In agreement with prior reports, we detected a large increase in proteins groups belonging to *Prevotella* after weaning (El-Deep et al., 2021; Minamida et al., 2005; Vasquez et al., 2022). The genome of *Prevotella* is enriched for gene families involved in the breakdown of plant-based carbohydrates introduced in the diet of weaned pigs (Minamida et al., 2005; Vasquez et al., 2022). In our data, *Prevotella* was one of the top CAZyme producers but no more so than other genera such as *Subdoligranulum*, *Sodaliphilus*, and *Bacteroides*, among others. The abundance of *Subdoligranulum* was positively associated with average daily growth in suckling and weaned piglets (Mahmud et al., 2023), therefore it is possible that production of CAZymes could contribute to improved growth via improving feed digestibility. Lastly, members of the phylum Bacillota including *Anaerobibrio*, *Lactobacillus* and *Mitsuokella*, produced the most abundant proteins associated with the metabolism of sucrose. The functional shifts recorded here highlight the flexibility of the gut microbiome in response to dietary changes, ensuring effective carbohydrate digestion and energy utilization.

### SCFA production and gut health

#### Acetate production

Following glycolysis, the metabolic end-product pyruvate undergoes fermentation in the hindgut to produce acetyl-CoA and ultimately SCFAs, the most common of which are acetate, propionate, and butyrate. Acetate production in piglets is crucial for maintaining intestinal barrier integrity, modulating gut microbiota, and supporting digestive health, particularly during the critical weaning period when gut function is developing (Shen et al., 2022; Holmes et al., 2020). Additionally, acetate plays a role in reducing gastrointestinal disease incidence, offering potential benefits in piglet health management and alternatives to antibiotic use (Holmes et al., 2020). Consistent with prior reports, members of the order *Eubacteriales* and *Bacteroidales*, especially Prevotellaceae, produced most of the proteins involved in pyruvate fermentation to acetate (Guevarra et al., 2019; Guevarra et al., 2018). In addition, microbes from the orders *Eubacteriales* and *Synergistales* produced acetate via the ethanolamine pathway in pre-weaned piglets. Although intestinal pathogens including enterotoxigenic *Escherichia coli* and *Salmonella enterica* metabolize ethanolamine (Lu et al., 2022; Brinsmade and Escalante-Semerena, 2004), this study suggests that microbes from diverse orders utilize phosphatidylethanolamine derived from the intestinal epithelium as a source of carbon and nitrogen (Garsin, 2010; Kaval and Garsin, 2018).

#### Propionate production

Propionate has a critical impact on swine health via the stimulation of satiety, protection against acute inflammation, and reduction of intestinal permeability (Vasquez et al., 2022; Zhang et al., 2022; Andrani et al., 2023; Gardiner et al., 2020). Generation of propionate occurs via three pathways, depending on the starting carbohydrate. These include the (1) succinate (hexose sugar); (2) propanediol (rhamnose and fucose sugars); and (3) acrylate (lactate intermediary) pathways (Figure 7). Formation of propionate via the succinate pathway is widely considered the dominant pathway (Reichardt et al., 2014; Deleu et al., 2021). Intriguingly, our results suggest that synthesis of propionate takes place predominantly via the propanediol pathway in pre-weaned pigs (Figures 4B, 6). Proteins associated with the fermentation of deoxy sugars (fucose, rhamnose) via the propanediol pathway were more abundant compared to those in the succinate pathway. This observation reflects a preference for the formation of propionate from available oligosaccharides such as fucose and rhamnose which are common in porcine milk and a major component of host-derived glycans (Wei et al., 2018). Post-weaning, propionate production shifted to the succinate pathway, more suited for metabolizing plant-derived carbohydrates. The transition between these pathways underscores the microbiome’s metabolic flexibility, ensuring the continuous production of SCFAs under different dietary conditions.

#### Butyrate production

Butyrate is essential for colonic health and energy supply. Functional assessment of pathways associated with butyrate production yielded no differences in the overall abundance before or after weaning. Of the taxa identified in pathway and terminal enzyme analyses, almost all were described prior in the literature to produce butyrate. Pathway analyses indicate that *Catenibacterium*, *Intestinimonas*, *Lachnospira*, and *Megasphaera* were among the most abundant butyrate producers in pre-weaned pigs, although only a single gene from the butanoate pathway was detected from *Lachnospira* and thus additional studies are needed to confirm a role in butyrate production for this taxon. Prior, *Megasphaera* was associated with butyrate production, especially under a high-fiber diet (Li et al., 2016). *Catenibacterium* and *Intestinimonas* are important butyrate producers in the human gut, with *Catenibacterium* playing a role in fiber fermentation and *Intestinimonas* contributing directly to butyrate production (Yan et al., 2013), but have not been well documented in pigs. *Lachnospira* has also been associated with butyrate production in the human gut (Vital et al., 2017). After weaning, the genera *Roseburia* and *Faecalibacterium* produced the highest abundance of proteins associated with the butanoate pathway. *Faecalibacterium* and *Roseburia* are involved in butyrate production in both humans (Barcenilla et al., 2000; Lopez-Siles et al., 2017) and pigs (Li et al., 2016; Uddin et al., 2023). The abundance of *Faecalibacterium* in the porcine gut is strongly correlated with increased fecal butyrate concentrations (Xu et al., 2021).

One limitation of the KEGG pathway analysis is that in some cases the enzymes present in the butanoate pathway are also involved in the generation of other SCFAs (e.g., acetate and propionate) or other metabolites. However, the terminal enzymes atoD, atoA, but, and buk are unique to the butyrate production and thus genera which produce them can be considered as active butyrate producers. These include *Romboutsia, Eubacterium, Anaerostipes, Pseudoramibacter, Roseburia,* and *Sodaliphilus*. Most of these genera belong to the class Clostridia (phylum: Bacillota), with the exception of *Sodaliphilus*, which is in the class *Bacteroidia* (phylum: Bacteroidota). *Eubacterium* is particularly notable for its dual butyrate production via both the acetyl-CoA and lysine pathways. While ample literature supports the butyrate-producing capacity of these genera, *Pseudoramibacter* and *Romboutsia* remain less studied, though our findings suggest they do produce butyrate through the acetyl-CoA and lysine pathways, respectively.

### Fungal roles and microbial cross feeding

While relatively little is known about the importance of fungi in non-ruminant animals, fungi are highly active in the microbiome of ruminant animals through the production of fiber-degrading enzymes such as cellulases and xylanases, which degrade carbohydrates that are indigestible to the host. In our analysis, fungi disproportionately exhibited carbohydrate degradation functions. Although the abundance of fungi in the microbiome is generally less than 0.1%, fungi accounted for ~8% of all CAZymes produced pre-wean, and ~3% of post-wean CAZymes (Supplementary Table S1). The single most abundant protein group involved in the degradation of the plant polysaccharide, xyloglucan, was produced by the fungi *Aspergillus*. Other substrates metabolized by proteins attributed to fungal taxa include α-glucan (*Talaromyces*), α-mannan and β-galactan (*Fusarium*), starch (Ascomycota), and host-glycans (*Candida* and *Aspergillus*). In the past, studies have shown a positive interaction between fungal microbes and nutrient digestibility, absorption, and intestinal health (El-Deep et al., 2021; Gordon and Phillips, 1998; Urubschurov et al., 2011). *Aspergillus* extract applied to feed as a prebiotic has been shown to promote nutrient absorption in growing pigs (den Besten et al., 2013). Somewhat unusually, fungal enzymes responsible for the breakdown of the plant substrates β-galactan, starch, and xylose were more abundant before weaning. Although plant-based feed is not directly fed to piglets, piglets routinely scavenge feed from the sow which could explain the presence of these enzymes before weaning. The abundances of all other fungal proteins in the study remained at similar levels before and after weaning except for those produced by *Candida* which were slightly more abundant after weaning. We speculate that enzymes synthesized by fungal microbes could aid in the transition from milk to solid feed in young piglets by improving feed digestibility.

We observed patterns consistent with microbial cross-feeding, where the products from one metabolic process are utilized by a different species or strain. This process enhances nutrient availability and can ensure efficient carbohydrate utilization, particularly during dietary transitions. Several microbial members produced proteins involved in the degradation of oligosaccharides but not glycolysis or SCFA production, suggesting a role as primary degraders (Mataigne et al., 2021). These include the genera: *Alistipes, Bifidobacterium* and *Muribaculum* (sucrose), *Talaromyces,* and *Muribaculum* (α-glucan), *Alistipes, Enterococcus*, and *Fusicatenibacter* (β-glucan), *Fusicatenibacter* and *Catenibacterium* (β-galactan), and *Pseudoramibacter* (starch). Only the fungus *Candida* displayed features of cross-feeding for the breakdown of host glycan substrates. Intriguingly, the abundance of proteins involved in cross-feeding largely remained stable at both timepoints, except for those produced by *Alistipes*. Of note, a large proportion (10/14) of all observed mucin and N-linked degrading enzymes were predicted to be exported to either the cell membrane or extracellular space. This suggests that breakdown of these carbohydrates occurs in a region that would make the subsequent by-product accessible to other microorganisms in the piglet gut lumen (Mataigne et al., 2021). Together, our observation suggests that cross-feeding and redundancy play a crucial role ensuring the maintenance of essential functions and system resiliency.

## Limitations and advantages of a metaproteomics approach

A proteomics approach, like other -omics methods, is not without limitations. In a typical experiment, proteins are extracted, digested into peptides, and analyzed via liquid chromatography coupled to tandem mass spectrometry (LC–MS/MS). Peptide spectra are then matched against in-silico peptide spectra predicted from database sequences, and proteins are inferred based on peptide identifications. Biases are introduced during each of these steps (Hettich et al., 2013; Van den Bossche et al., 2021). A few of the most significant factors include protein extraction efficiencies (Zhang et al., 2018), liquid chromatography, MS/MS fragmentation, and database composition. In addition, environmental samples contain many proteins from dozens to hundreds of taxa, and this complexity can exacerbate these issues. Thus, it is likely that some proteins may not have been detected or identified, particularly those which are lowly abundant or specific to certain species and strains.

Nonetheless, metaproteomics offers several unique advantages. Most importantly, this technique elucidates active proteins, leading to a comprehensive understanding of microbiome functions. For example, we detected and identified proteins associated with SCFA expression among some taxa previously predicted to synthesize SCFAs, including the genera *Clostridium*, *Intestinimonas, Ruminococcus*, *Prevotella*, *Bacteroides*, *Fusobacterium*, *Bifidobacterium, Blautia, Dialister, Faecalibacterium,* and *Acidaminococcus* (Long et al., 2020; Yang et al., 2022). However, we did not find evidence of SCFA production for other genera which have been predicted to synthesize SCFAs, although they were present in this dataset. An example of this is the genus *Akkermansia*. Although we did detect *Akkermansia* proteins, none were associated with SCFA production. This highlights the importance of monitoring protein production among gut microbial members, as (meta)genomic data alone does accurately describe all microbial functions.

### Implications for weaning management and closing remarks

In this study, we provide the first description of the fecal metaproteome of young piglets before and after weaning and demonstrate the role of the gut microbiome in carbohydrate digestion. These data provide a foundation for the development of practical solutions to improve piglet health and productivity. Promoting beneficial bacteria such as *Mitsuokella*, *Anaerovibrio*, *Prevotella*, *Blautia*, *Acidaminococcus*, *Bacteroides*, *Eubacterium*, *Subdoligranulum*, and *Catenibacterium* to increase production of beneficial short-chain fatty acids (SCFAs) and the degradation of dietary oligosaccharides could be achieved through tailored prebiotic or probiotic interventions. In addition, fungal therapeutic targets such as *Aspergillus* may prove valuable in the enhancement of feed digestibility, as fungi synthesized a large proportion of CAZymes in the microbiome. Furthermore, we discovered a pivotal shift in propionate production pathways, from the propanediol pathway in pre-weaned pigs to the succinate pathway post-weaning, offering potential targets for modulating SCFA synthesis and improving gut health. Developing therapeutics that manipulate these metabolic pathways at the appropriate time may help mitigate post-weaning growth lags and enhance overall performance.

## Data Availability

The original contributions presented in this study are publicly available. Sequencing data can be found in the NCBI repository under accession number PRJNA1020867, and proteomics data are available via ProteomeXchange with identifier PXD063155.
