# Supplementary figures and images for: A metaproteomic analysis of the piglet fecal microbiome across the weaning transition
Source: Front Microbiol. 2025 May 2;16:1504433. doi: 10.3389/fmicb.2025.1504433 (PMC12082470; doi:10.3389/fmicb.2025.1504433)

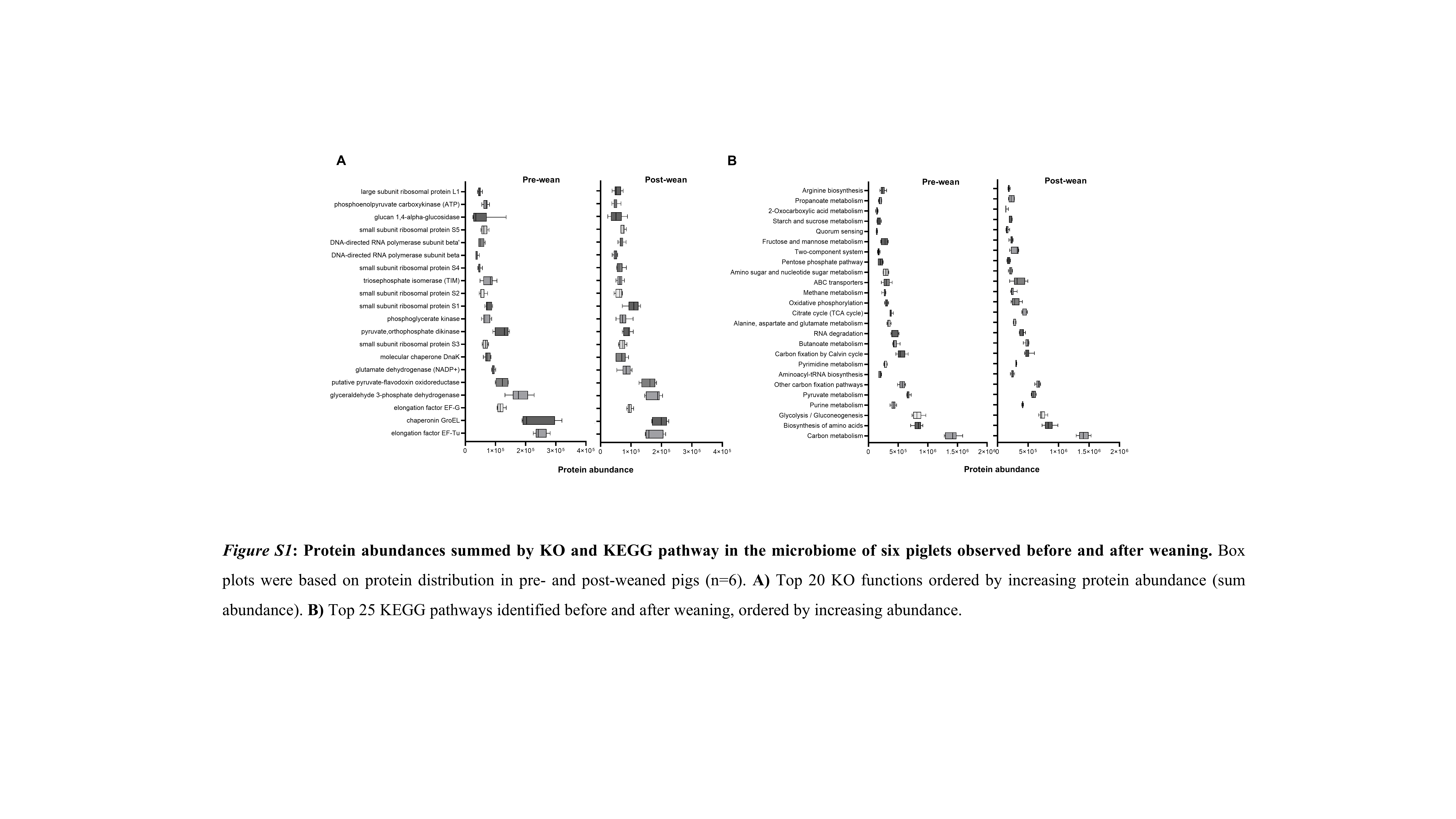

Supplement: Supplementary file 1 [file Image_1.tif]

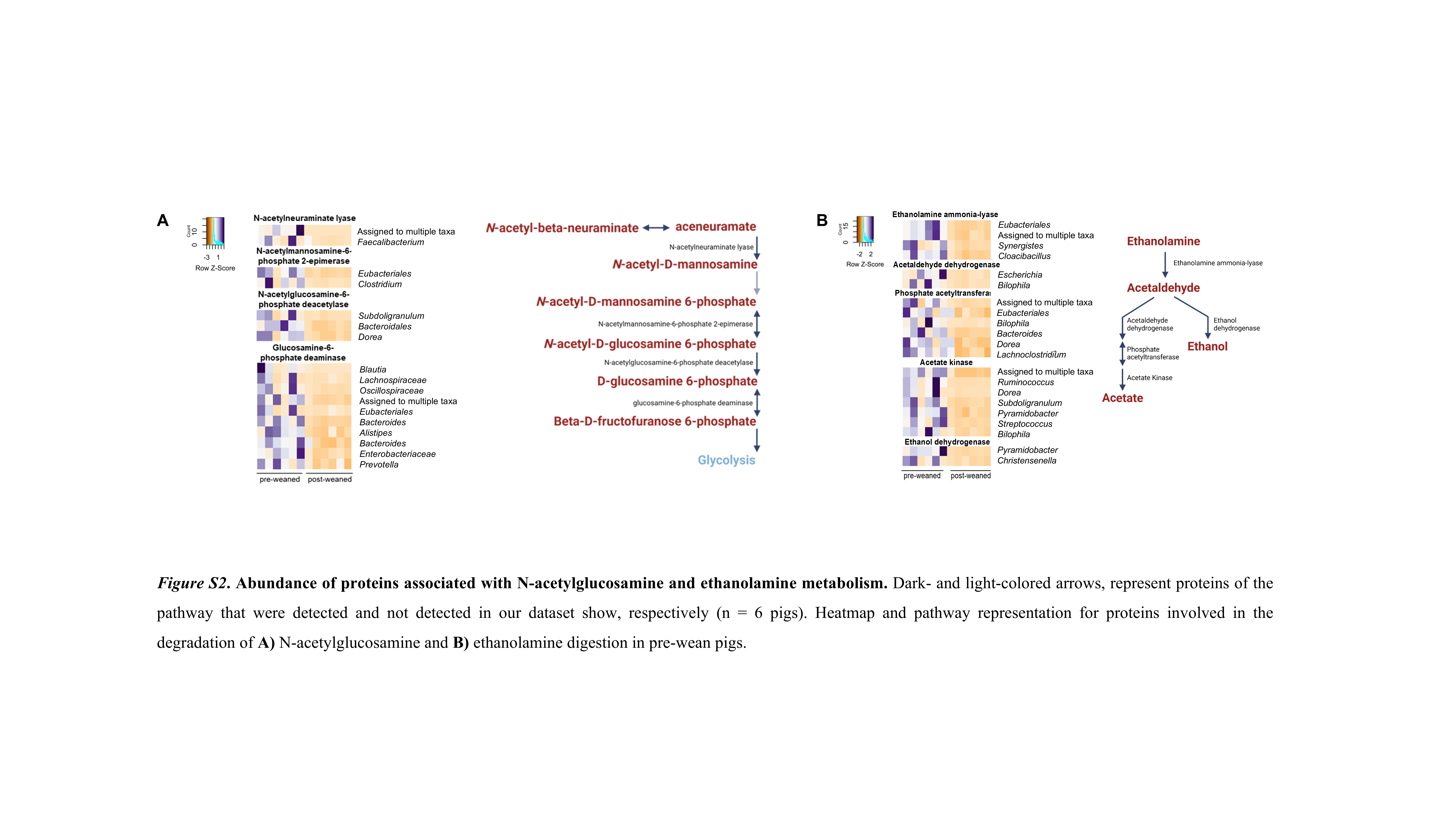

Supplement: Supplementary file 2 [file Image_2.tif]
